# Towards hybrid pixel detectors for energy-dispersive or soft X-ray photon science

**DOI:** 10.1107/S1600577515023541

**Published:** 2016-02-10

**Authors:** J. H. Jungmann-Smith, A. Bergamaschi, M. Brückner, S. Cartier, R. Dinapoli, D. Greiffenberg, T. Huthwelker, D. Maliakal, D. Mayilyan, K. Medjoubi, D. Mezza, A. Mozzanica, M. Ramilli, Ch. Ruder, L. Schädler, B. Schmitt, X. Shi, G. Tinti

**Affiliations:** a Paul Scherrer Institute, 5232 Villigen PSI, Switzerland; b Institute for Biomedical Engineering, University and ETHZ, 8092 Zürich, Switzerland; c Synchrotron Soleil, L’Orme des Merisiers, BP 48, Saint-Aubin, 91192 GIF-sur-Yvette Cedex, France

**Keywords:** hybrid detectors, soft X-rays, energy-dispersive detectors, instrumentation for FELs, instrumentation for synchrotrons

## Abstract

JUNGFRAU (adJUstiNg Gain detector FoR the Aramis User station) is a two-dimensional hybrid pixel detector for photon science applications at free-electron lasers and synchrotron light sources. The JUNGFRAU 0.4 prototype presented here is specifically geared towards low-noise performance and hence soft X-ray detection. The design, geometry and readout architecture of JUNGFRAU 0.4 correspond to those of other JUNGFRAU pixel detectors, which are charge-integrating detectors with 75 µm × 75 µm pixels. Main characteristics of JUNGFRAU 0.4 are its fixed gain and r.m.s. noise of as low as 27 e^−^ electronic noise charge (<100 eV) with no active cooling. The 48 × 48 pixels JUNGFRAU 0.4 prototype can be combined with a charge-sharing suppression mask directly placed on the sensor, which keeps photons from hitting the charge-sharing regions of the pixels. The mask consists of a 150 µm tungsten sheet, in which 28 µm-diameter holes are laser-drilled. The mask is aligned with the pixels. The noise and gain characterization, and single-photon detection as low as 1.2 keV are shown. The performance of JUNGFRAU 0.4 without the mask and also in the charge-sharing suppression configuration (with the mask, with a ‘software mask’ or a ‘cluster finding’ algorithm) is tested, compared and evaluated, in particular with respect to the removal of the charge-sharing contribution in the spectra, the detection efficiency and the photon rate capability. Energy-dispersive and imaging experiments with fluorescence X-ray irradiation from an X-ray tube and a synchrotron light source are successfully demonstrated with an r.m.s. energy resolution of 20% (no mask) and 14% (with the mask) at 1.2 keV and of 5% at 13.3 keV. The performance evaluation of the JUNGFRAU 0.4 prototype suggests that this detection system could be the starting point for a future detector development effort for either applications in the soft X-ray energy regime or for an energy-dispersive detection system.

## Abstract 2

A novel hybrid pixel detector is evaluated and its potential for low-noise/low-energy detection and energy-dispersive photon science is highlighted.

## 1. Introduction   

New generations of X-ray sources, both free-electron lasers and synchrotrons, are currently being designed, built and commissioned or upgraded (Patterson *et al.*, 2010 ▸; Pile, 2011 ▸; Arthur *et al.*, 1995 ▸, 2012 ▸; EU-XFEL, 2015 ▸; Admans *et al.*, 2014 ▸; Ehrlichman *et al.*, 2014 ▸). These light sources will be increasingly powerful, brilliant and versatile in their performance characteristics. This results in a diverse demand for adequate imaging detection systems for photon science at these facilities.

The main performance criteria for these radiation detectors (Graafsma, 2009 ▸) comprise, but are not limited to, single-photon sensitivity, a good linearity of response (linear to within a few percent) over a dynamic range of ten thousands of photons per frame per pixel, and frame rates adequate to the machine repetition rates and/or expected photon fluxes. Geometrical requirements range from two-dimensional (pixel) detection systems, small pixel sizes of the order of less than 100 µm [similar to state-of-the-art photon-counting detectors and towards charge-coupled device (CCD) pixel sizes] to modular, tilable, vacuum-compatible imaging detectors. Current and future detector demands for the new X-ray free-electron laser (XFEL) and synchrotron sources are being met and anticipated by several ongoing detector developments (Bergamaschi *et al.*, 2015 ▸).

One of the future directions of these novel light sources is the extension of their capabilities to the soft X-ray regime, *i.e.* the energy range below 5 keV (Patterson *et al.*, 2010 ▸; Patterson & Van Daalen, 2014 ▸; Arthur *et al.*, 2012 ▸). One envisioned project is the ATHOS soft X-ray beamline (Patterson & Van Daalen, 2014 ▸) of SwissFEL which would enable photon science experiments in the X-ray energy range from 180 eV to 1.8 keV. Concerning detector technology, this domain is at present primarily served by charge-coupled device (CCD) cameras, monolithic active pixel sensors (MAPS) for imaging applications. Applications using single detection channels are mainly covered by silicon drift detectors (SDD) or avalanche photodiodes (APD), which feature a relatively high energy and time resolution, respectively.

Currently, several general purpose detector systems sensitive to photons in the energy range from a few hundred eV to several tens of keV are under development (Wunderer *et al.*, 2014 ▸; Meidinger *et al.*, 2006 ▸; Dinapoli *et al.*, 2014 ▸; Mozzanica *et al.*, 2014 ▸). Jointly, these developments fight the challenge of achieving a low probability of false-positive photon counts in the soft X-ray energy range, which translates into the need for a low noise level, *i.e.* noise on the level of a few tens of electrons (e^−^) or less. Selected general purpose detector developments include among others the PERCIVAL (Pixelated Energy Resolving CMOS Imager, Versatile and Large) (Wunderer *et al.*, 2014 ▸) system. PERCIVAL is a back-thinned MAPS designed to target photon detection in the energy range from 250 eV to 1 keV with a quantum efficiency of 90% where a small input node capacitance and an operating temperature between 243 K and 233 K help to minimize the noise of the system. PERCIVAL will feature 3520 × 3710 pixels with a 27 µm pitch on an area of 10 cm × 10 cm. Multiple gains cover a dynamic range of 1–10^5^ simultaneously arriving photons (at 500 eV) at a readout rate of 120 Hz. Another detection system for the lower photon energy range is the pnCCD (Meidinger *et al.*, 2006 ▸), which has a root-mean-square (r.m.s.) noise of 60 e^−^ in the high-frame-rate version used in photon science applications. The pnCCD consists of a 256 × 256 pixel matrix of 75 µm × 75 µm pixels, is operated at a temperature of 248 K and runs at a frame rate of 120 Hz. Lastly, MÖNCH 0.3 (Dinapoli *et al.*, 2014 ▸) is a hybrid pixel detector with 400 × 400 pixels of 25 µm × 25 µm each (10 mm × 10 mm active area), which features 30 e^−^ noise (for MÖNCH 0.2) (Bergamaschi *et al.*, 2015 ▸), a ∼6 kHz readout rate and a very high spatial resolution of a few micrometers *via* offline analysis of the charge-sharing clouds.

Besides these general purpose detector developments, also a few dedicated energy-dispersive systems aim to advance fluorescence/elemental spectroscopy and microscopy applications at existing and future synchrotron and XFEL beamlines (Siddons *et al.*, 2014 ▸; Kenny, 2015 ▸). One of these developments is the MAIA detector (Siddons *et al.*, 2014 ▸) for energy-dispersive fluorescence spectroscopy and elemental imaging, which consists of 384 diode elements of 1 mm^2^ each and comprises a three-layer absorber geometry to prevent charge sharing. MAIA is operated at 238 K and handles photon rates of up to 10^7^ photons s^−1^ on the detector array. An interesting new development for spectroscopic applications in the soft X-ray energy range is the ePixS (Kenny, 2015 ▸). ePixS is characterized by a r.m.s. noise of 8 e^−^ (no sensor), 500 µm × 500 µm pixels, two auto-ranging gains and frame rates up to 20 kHz.

The JUNGFRAU (adJUstiNg Gain detector FoR the Aramis User station) hybrid pixel detector (Mozzanica *et al.*, 2014 ▸; Jungmann-Smith *et al.*, 2014 ▸), in particular the low noise prototype JUNGFRAU 0.4, could be a starting point for either a low-energy general purpose detection system or an energy-dispersive detection system for spectroscopic applications. In this paper, the JUNGFRAU hybrid pixel detector, in particular the low-noise prototype JUNGFRAU 0.4, will be introduced (§2). The characterization of JUNGFRAU 0.4 in terms of noise, gain and dynamic range and its implications for low-energy photon science applications will be presented in §3. §4 shows first low-energy measurements, multi-photon-counting spectra and establishes the photon energy acceptance range, energy resolution and pixel linearity. §5 presents energy-dispersive measurements from X-ray tube and synchrotron light irradiation and comments on obtainable photon rate capabilities. The manuscript concludes by highlighting the capabilities added by this new detection system and provides an outlook on future opportunities for low-energy or energy-dispersive detectors based on JUNGFRAU 0.4 (§6).

## 2. The JUNGFRAU hybrid pixel detector   

### 2.1. Design characteristics, geometry and readout   

JUNGFRAU is a hybrid pixel detector for photon science applications at free-electron lasers and synchrotron light sources (Mozzanica *et al.*, 2014 ▸; Jungmann-Smith *et al.*, 2014 ▸). Major characteristics of the JUNGFRAU charge-integrating pixel detector are single-photon sensitivity, and a low noise performance over a dynamic range of 10^4^ 12 keV photons. These specifications are enabled by a gain-switching (three gains) preamplifier in each pixel, which dynamically adjusts its gain to the amount of charge deposited on the pixel, similar to AGIPD (Göttlicher *et al.*, 2009 ▸) or GOTTHARD (Mozzanica *et al.*, 2012 ▸). Performance characteristics of the JUNGFRAU chips also include a linearity of response and gain uniformity (linear to within few percent) and spatial resolving power similar to single-photon detectors with the same pixel pitch (Mozzanica *et al.*, 2014 ▸; Jungmann-Smith *et al.*, 2014 ▸).

Geometrically, a JUNGFRAU 1.0 chip measures about 2 cm × 2 cm and comprises 256 × 256 pixels of 75 µm × 75 µm each. The chips are bump-bonded to 320 µm silicon sensors. The quantum efficiency of standard silicon sensors decreases rapidly below incident photon energies of 1.5 keV, which calls for the development of sensors with thinner entrance windows for low photon energy applications. Specifically, the quantum efficiency of the JUNGFRAU sensor is about 60% (20%) for photon energies of 1 keV (500 eV) and could be improved to about 85% (80%) for a sensor with a thinner entrance window (as computed from the nominal thicknesses of the implant and passivation layers provided by the sensor manufacturer). 2 × 4 chips are tiled to form modules of 4 cm × 8 cm. Several multi-module systems with up to 16 Mpixels per system are planned for the two end-stations at SwissFEL and synchrotron beamlines. The anticipated readout rate in excess of 2 kHz is independent of the detector size and enables a dead-time-free linear count rate capability of 20 MHz pixel^−1^ (50 MHz pixel^−1^) for 12 keV (5 keV) photons, which meets both the readout requirements of SwissFEL and high-count-rate synchrotron experiments.

JUNGFRAU provides the community with a comparable data quality to single-photon-counting systems. This, in combination with its specifications and performance characteristics, makes this charge-integrating readout chip an attractive detection system for efficient detection at both free-electron lasers and synchrotron light sources [details are given by Mozzanica *et al.* (2014 ▸) and Jungmann-Smith *et al.* (2014 ▸)].

### 2.2. JUNGFRAU 0.4   

The JUNGFRAU 0.4 prototype is a variation of JUNGFRAU, which is specifically dedicated to low-noise performance. The geometry and the pixel layout of JUNGFRAU 0.4 are equivalent and fully compatible to the mechanics and the readout of other JUNGFRAU chips. JUNGFRAU 0.4 is modified by few adaptations in the pixel circuit. In particular, the circuit is equipped with a fixed and increased gain (as compared with gain switching in other JUNGFRAU variations). The superfluous gain-switching capacitors can optionally be connected to the preamplifier output as filtering capacitors, which reduce the pixel noise. The rise time of the preamplifier is 175 ns (without additional filtering) and 525 ns (with additional filtering), respectively. In addition, the preamplifier layout is optimized to reduce the parasitic input capacitance. At present, a 48 × 48 pixels prototype chip of JUNGFRAU 0.4 (3.6 mm × 3.6 mm) bump-bonded to a 500 µm-thick silicon sensor is under evaluation (sensor choice due to availability).

### 2.3. Charge-sharing suppression: JUNGFRAU 0.4 with a hole mask   

**Figure 1:**
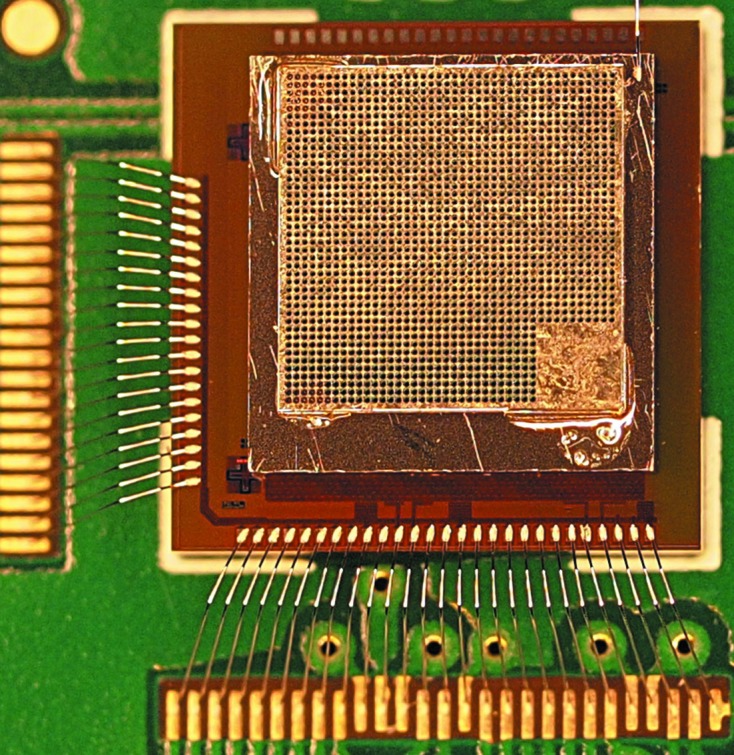
Photograph of the JUNGFRAU 0.4 chip and sensor on top of which a 150 µm-thick laser-drilled tungsten mask (Laser Zentrum Hannover eV, Hannover, Germany) with ∼28 µm-diameter holes is placed. The chip and sensor are about 3.6 mm × 3.6 mm in size, while the mask dimensions are about 3.3 mm × 3.3 mm. The total active area in the mask region is about 1.1 mm^2^. The mask is placed on top of the sensor and the mask holes are aligned with the center of the pixels with an automatic sub-micrometer die bonder (Fineplacer Femto, Finetech GmbH & Co. KG, Berlin, Germany). The mask is attached with a drop of glue at each mask corner. The bottom right corner of the mask does not contain any holes for reference purposes.

JUNGFRAU 0.4 can be combined with a charge-sharing suppression mask. The hole mask is aligned with the pixel matrix and placed on top of the sensor of a hybridized JUNGFRAU 0.4 (Fig. 1 ▸). The alignment of the holes of the mask with the center of the pixels enables the passage of photons centered on the pixel cell, while the material of the mask attenuates photons in the charge-sharing region of the pixels and prevents the division of charge between multiple detector elements similar to Siddons *et al.* (2014 ▸) (see §4 and §5). Since charge sharing between multiple pixels can merge the noise peak and low-energy photon peaks, the suppression of charge sharing results in cleaner spectra particularly in the low-energy range where the noise and photon peaks can be overlapping in charge-integrating detection systems. The mask specifically removes the need to sum the signal of several pixels to obtain the full charge, which increases the noise. The mask, however, reduces the detection efficiency to π*r* ^2^/*p* ^2^, *i.e.* π(14 µm)^2^/(75 µm)^2^ = 0.11, where *r* = 14 µm is the radius of the mask holes and *p* = 75 µm is the JUNGFRAU pixel pitch. The hole diameter of ∼28 µm represents the exit diameter of the conical laser-drilled mask holes. A hole diameter of ∼28 µm is chosen for this first mask for technical production reasons and larger hole diameters defined by the charge-sharing distance of the pixels (Bergamaschi *et al.*, 2015 ▸) are planned for future experiments.

## 3. Characterization results   

### 3.1. Noise performance: implications for low-energy detection   

The noise performance of the JUNGFRAU 0.4 application-specific integrated circuit (ASIC) is evaluated for an acquisition time of about 2 µs, a typical value for X-FEL applications. Unless stated otherwise, no active cooling is applied to the detector assembly, which is operated at a constant temperature of about 30°C. The sensor is biased at 240 V. Noise measurements are performed in the absence of photon irradiation. Prior X-ray fluorescence irradiation measurements are employed to establish the gain of each pixel in units of eV ADC^−1^ (ADC = analog-to-digital converter units). The relationship of 3.6 eV per electron–hole pair in silicon sensors is used to express the noise performance of the ASIC in terms of the equivalent noise charge in units of e^−^. The pixel noise is evaluated as the variance of a Gaussian fit to the noise peak, *i.e.* a fit to the pixel output signal in the absence of photon signal.

**Figure 2:**
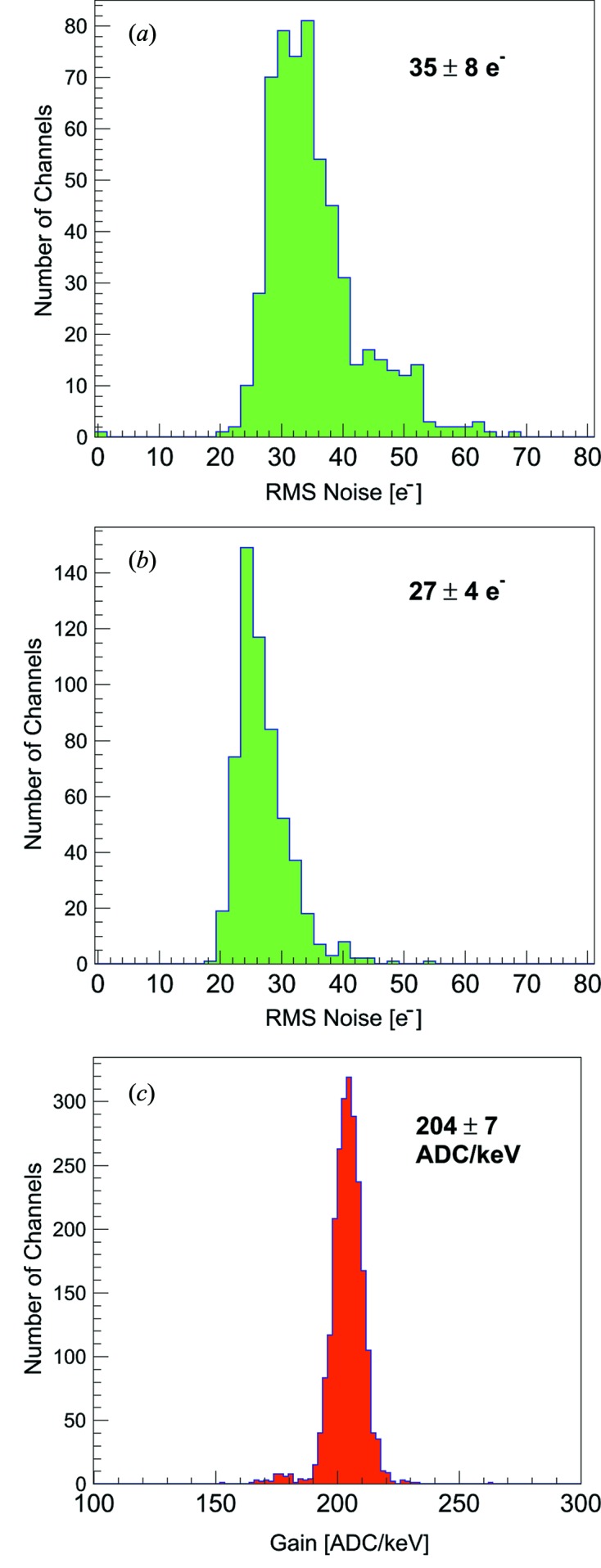
Distribution of r.m.s. noise for JUNGFRAU 0.4 (*a*) with no additional noise filtering and (*b*) with noise filtering. (*c*) Gain distribution of JUNGFRAU 0.4.

Fig. 2 ▸ displays the noise distribution of the JUNGFRAU 0.4 ASIC. The mean of the noise distribution for JUNGFRAU 0.4 is at an electronic noise charge (e.n.c.) of 35 ± 8 e^−^ for the unfiltered mode of operation. The noise distribution comprises pixels in a tail towards higher r.m.s. noise (40–65 e^−^). For the filtered mode, the mean of the noise distribution is at an e.n.c. of 27 ± 4 e^−^ and only very few pixels show a higher noise (>30 e^−^). The main contribution to the pixel noise is given by the pixel preamplifier, *i.e.* about 30 e^−^ for no filtering and about 21 e^−^ for extra filtering (Table 1 ▸). The readout chain and the correlated double sampling stage equally share the remaining noise contributions of about 12 e^−^, which are measured by putting the preamplifier or the CDS stage in reset.

The pixel r.m.s. noise of 27 e^−^ corresponds to about σ = 27 e^−^ × 3.6 eV/e^−^ = 97.2 eV in silicon sensors (Table 1 ▸). This means that this noise peak extends to about 300 eV (3σ = 291.6 eV) in the photon energy spectrum. This noise performance enables the JUNGFRAU 0.4 detection system to measure photon signal as low as ∼0.5 keV when assuming a 5σ statistical margin (5σ = 486 eV).

**Figure 3:**
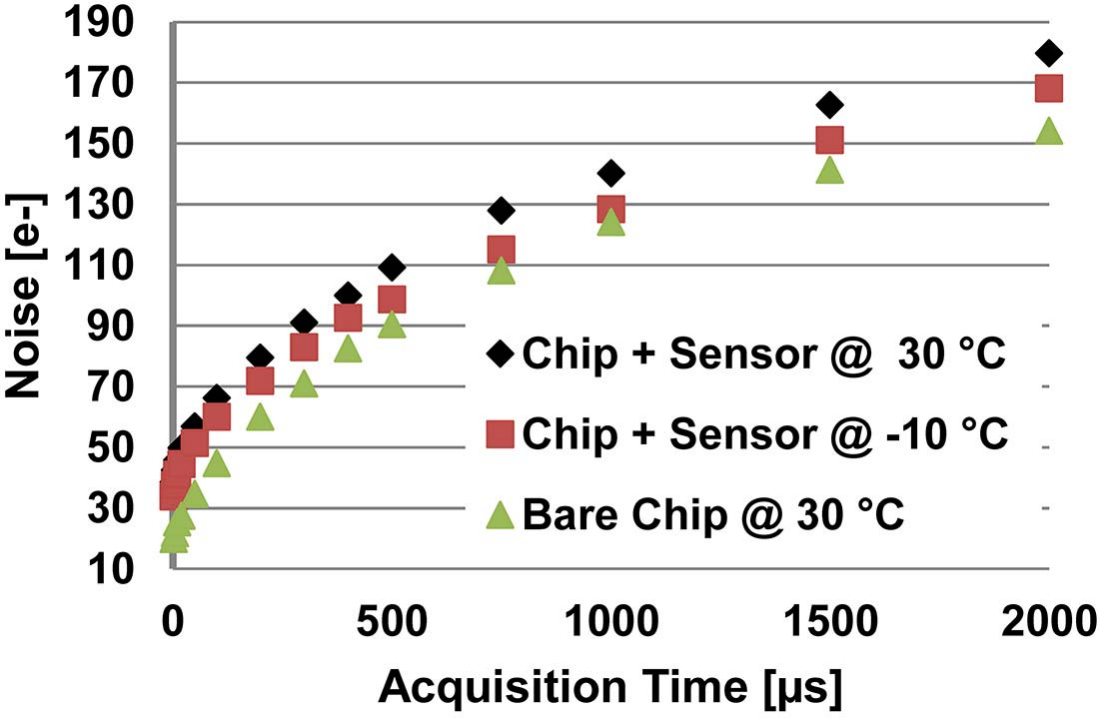
Mean of the r.m.s. noise of JUNGFRAU 0.4 (no extra filtering) as a function of the acquisition time for a hybridized assembly (chip bump-bonded to sensor) at chip operating temperatures of 30°C and −10°C and for a bare chip at a chip operating temperature of 30°C.

The r.m.s. noise of JUNGFRAU 0.4 can be reduced further by actively cooling the detector system (Fig. 3 ▸). At a detector operating temperature of −10°C, the noise of a hybridized assembly is reduced by about 12% for an acquisition time of 2 µs and by about 7% for an acquisition time of 2 ms.

The dependence of the JUNGFRAU 0.4 noise on the acquisition time is evaluated in the range from 2 µs (typical XFEL acquisition time) to 2 ms for a hybridized assembly at chip operating temperatures of 30°C and −10°C, respectively, and for a bare chip at an operating temperature of 30°C (Fig. 3 ▸). For the hybridized assembly, the r.m.s. noise gradually increases from the <35 e^−^ noise level for an acquisition time of 2 µs to about 180 e^−^ or 170 e^−^, respectively, for an acquisition time of 2 ms. For the bare chip, the noise increases from about 20 e^−^ at an acquisition time of 2 µs to about 155 e^−^ at an acquisition time of 2 ms. This shows that the increase in the noise level originates from two sources as expected: an increase in the sensor leakage current as indicated by the measurements with the hybridized assembly at 30°C and −10°C (sensor current not monitored during the measurement), and a different response of the noise filtering in the correlated double sampling stage as a function of acquisition time as exemplified by the bare chip measurement.

The JUNGFRAU 0.4 noise performance of <35 e^−^ represents a significant improvement in the noise performance as compared with previous JUNGFRAU iterations, *i.e.* the JUNGFRAU 0.2 has a r.m.s. noise of 100 e^−^ (Jungmann-Smith *et al.*, 2014 ▸). Additionally, the presence of random telegraphic noise signal (Card & Mavretic, 1963 ▸) is not observed in JUNGFRAU 0.4.

**Figure 4:**
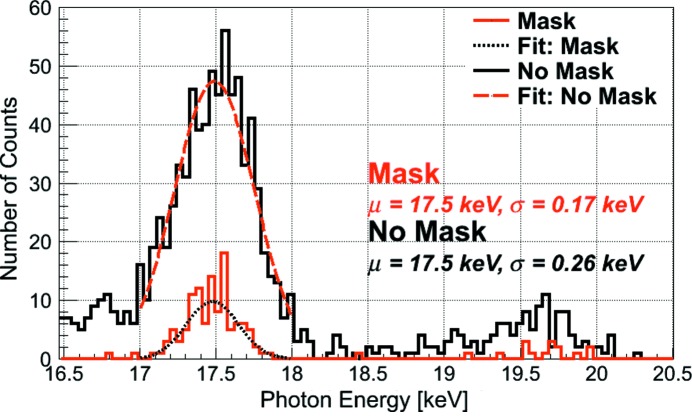
Fluorescence spectra of Mo from single JUNGFRAU 0.4 pixels with the mask and without the mask. The noise of the JUNGFRAU 0.4 pixel can be determined from the spectrum of the masked pixel due to the absence of charge-sharing counts in the spectrum and is determined to be 48 e^−^.

In combination with the mask, the noise of JUNGFRAU 0.4 can be determined from any isolated, monochromatic photon peak since the mask removes the charge sharing between pixels and hence the peak width is determined by the pixel noise only (Fig. 4 ▸). The r.m.s. noise of a randomly selected masked JUNGFRAU 0.4 pixel at an acquisition time of 20 µs is, for instance, found to be about 48 e^−^ from the standard deviation of a Gaussian fit to the *K*_α_(Mo) peak (description of measurement in §5.1), which is in accordance with the noise measurements presented in Fig. 3 ▸. A Gaussian fit to an unmasked pixel returns a standard deviation of about 73 e^−^, which is due to the charge-sharing tail forcing the Gaussian fit to be wider.

### 3.2. Gain and dynamic range   

The preamplifier gain of JUNGFRAU 0.4 is fixed and is independent of the applied noise filtering in the circuit. The chip is calibrated with X-ray irradiation from fluorescence samples, which results in a gain of 204 ADC keV^−1^ with a spread in the gain values of <3.5% throughout the pixel matrix (Fig. 2*c* ▸).

The dynamic range of JUNGFRAU 0.4 at a given photon energy is estimated by the gain, the ADC range of 16384 ADC units and the pixel offset. The dynamic range is about 49, 10 or 4 photons for 1 keV, 5 keV and 12 keV photons, which represents an adequate dynamic range for fluorescence measurements at synchrotron and FEL light sources.

## 4. Measurements at low photon energies   

### 4.1. Spectra at low energies: with and without charge sharing suppression   

The low-energy performance of JUNGFRAU 0.4 is evaluated in the energy range from 1.2 to 2.0 keV at the PHOENIX beamline of the Swiss Light Source, Paul Scherrer Institut, Villigen, Switzerland. The JUNGFRAU 0.4 detector assembly printed-circuit board is mounted and operated in-vacuum (∼10^−3^ mbar) while held at a constant temperature of 15°C by active cooling. The silicon sensor is biased at a voltage of 400 V to obtain a better response for these low-energy measurements, *i.e.* the increase in the bias voltage slightly extends the depleted region into the shallow n+ backplane implant which reduces the dead volume of the entrance window. The readout electronics system is placed outside the vacuum chamber. The detector assembly and the readout system are connected by 50 cm-long one-to-one flat ribbon cables that are introduced into the vacuum chamber through a custom feedthrough. It is established experimentally that the performance and data quality of JUNGFRAU 0.4 is not affected by the introduction of these extension cables.

**Figure 5:**
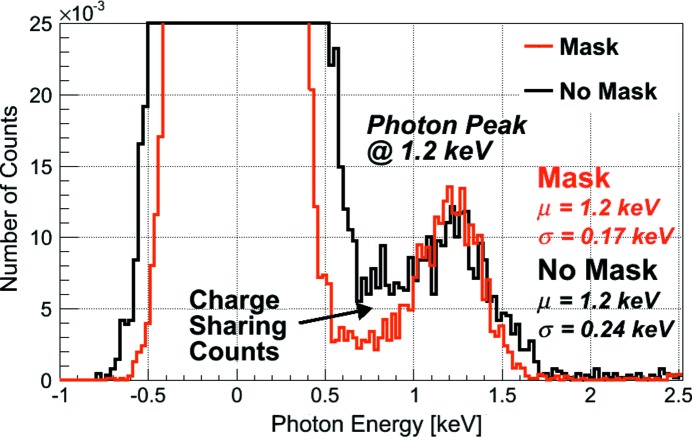
JUNGFRAU 0.4 single-pixel spectra at a photon energy of 1.2 keV. The normalized spectrum of a pixel from a detector without a mask (black) and the spectrum of a pixel from a detector with a hole mask (red) are superimposed.

Fig. 5 ▸ displays the spectra of a single masked and a single non-masked JUNGFRAU 0.4 pixel when flat-field illuminated at a photon energy of 1.2 keV. The pixels are chosen randomly, *i.e.* there is no selection based on the pixel noise. The normalized spectra of an unmasked JUNGFRAU 0.4 pixel (acquisition time of 5 µs frame^−1^, accumulated for ∼97 kframes) and of a masked pixel (acquisition time of 2 µs frame^−1^, accumulated for ∼95 kframes) are compared, *i.e.* a pixel for which the charge sharing is suppressed by the tungsten mask. The charge sharing is reduced significantly by the placement of the mask. A Gaussian fit to the 1.2 keV photon peaks of the normalized spectra determines the full width at half-maximum (FWHM) of the peaks to be about 570 eV and 400 eV for the unmasked and masked assemblies, respectively. The Gaussian fit to the unmasked pixel is wider (compared with the masked pixel) due to the charge-sharing tail forcing the fit to be wider and also the longer acquisition time. This corresponds to a Δ*E*/*E* of 0.20 and 0.14, respectively.

### 4.2. Multi-photon spectra at low energies   

**Figure 6:**
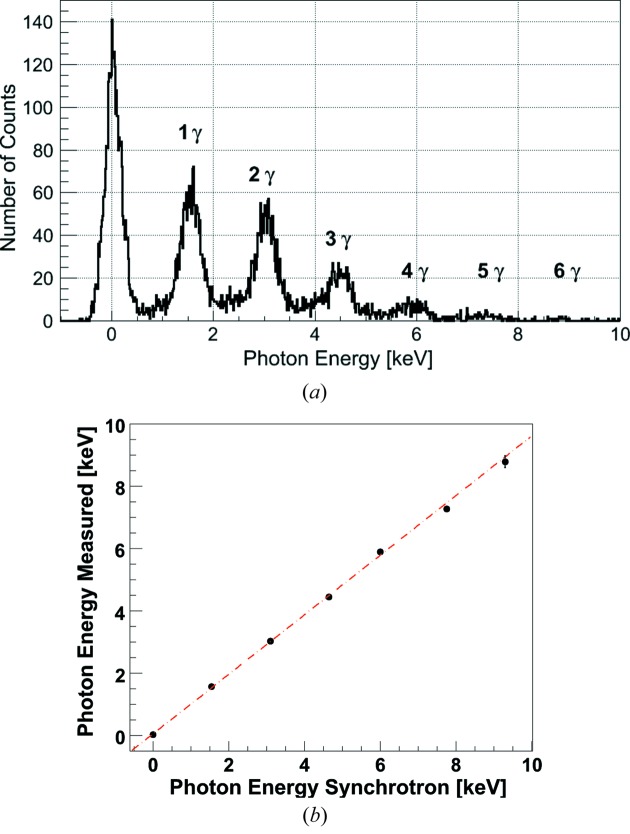
(*a*) JUNGFRAU 0.4 multi-photon spectrum of a single pixel at a single photon energy of 1.55 keV. (*b*) Linear pixel response.

A single-pixel spectrum with multiple 1.55 keV photons is recorded by JUNGFRAU 0.4 in combination with the charge-sharing suppression mask (Fig. 6*a* ▸). The acquisition time is 5 µs frame^−1^ and about 8 kframes are recorded. Fig. 6 ▸ demonstrates that monochromatic photons can be ‘counted’ with a charge-integrating hybrid pixel detector like JUNGFRAU. In this particular example, up to six photons of 1.55 keV energy each are counted per pixel. Fig. 6(*b*) ▸ shows that the pixel response is linear within the energy range from 0 to 10 keV where additional uncertainty might have been introduced by relatively lower statistics for the higher multi-photon peaks.

## 5. Multi-color X-ray measurements   

### 5.1. Energy-dispersive photon measurements and charge-sharing suppression   

**Figure 7:**
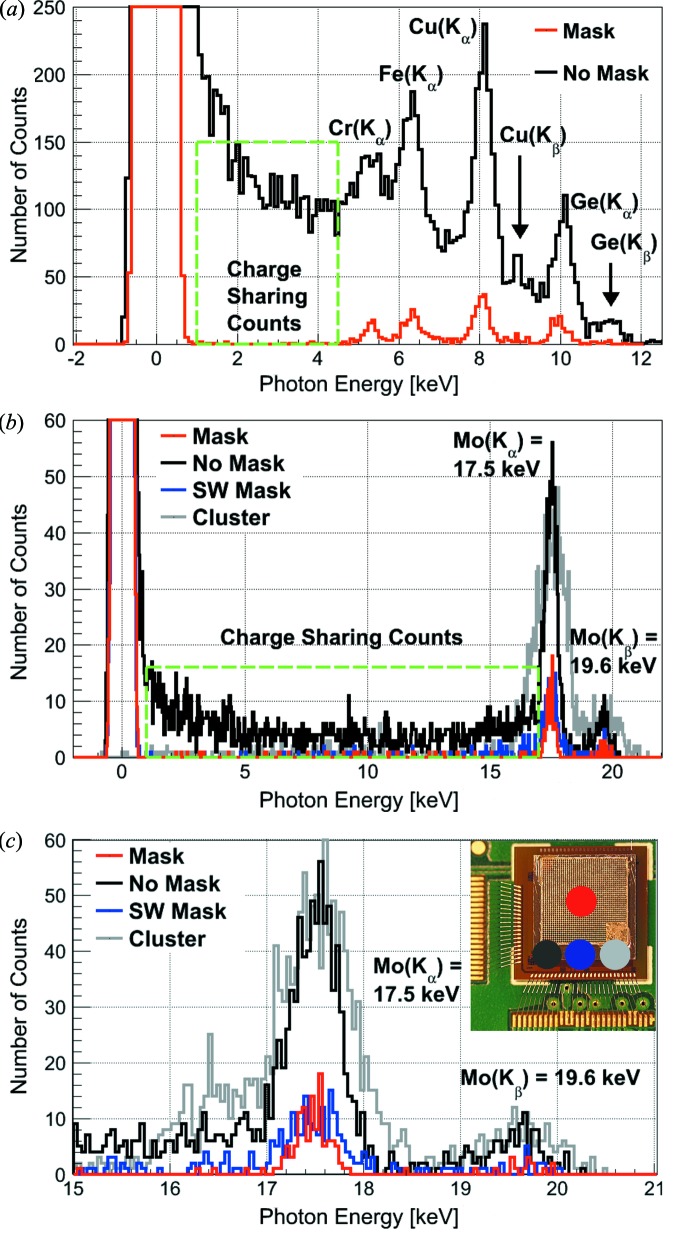
Single-pixel fluorescence X-ray spectra acquired by JUNGFRAU 0.4 with and without the charge-sharing suppression mask. (*a*) Energy-dispersive measurements of fluorescence photons from a composite Cr, Fe, Cu and Ge target. (*b*) Fluorescence spectrum from a Mo target. The spectra of a masked (red) and an unmasked pixel (black) are superimposed for direct comparison; the spectra obtained from the software mask (blue) and the cluster-finding algorithm (gray) applied to the data of the unmasked pixel are included for the Mo fluorescence (*b*). (*c*) Zoom on the Mo peaks.

JUNGFRAU 0.4 (in combination with the charge-suppression mask) is employed as an energy-dispersive detection system. Fig. 7(*a*) ▸ shows single-pixel spectra of a multi-color fluorescence X-ray experiment acquired with a Cu X-ray tube setup (General Electric Company, Seifert ID 3003 and analytical X-ray tube, 30 kV, 60 mA) and a multi-color target composed of Cr, Fe, Cu and Ge rods. The measurements are performed at a source-to-detector distance of about 35 cm to reduce the effect of parallax on the mask geometry. An acquisition time of 20 µs (∼890 kframes) is used.

The spectrum of an unmasked pixel is shown in black, while the spectrum of a pixel situated underneath the charge-suppression mask is displayed in red as schematically indicated by the red and black circles in the inset of Fig. 7(*c*) ▸. The *K*_α_ lines of Cr, Fe, Cu and Ge are resolved in both spectra and traces of the *K*_β_ lines of Cu and Ge are observed. The peak-to-valley ratio is significantly higher in the masked pixel spectrum due to the removal of the charge-sharing contribution, *i.e.* the peak-to-valley ratio for the Cu(*K*_α_) peak of a pixel situated underneath the mask is 35 *versus* 3.5 for an unmasked pixel. The mask removes the charge-sharing contribution to the spectrum, which is present in the energy range from 750 eV to 4.5 keV in the spectrum of the unmasked pixel and absent in the spectrum of the masked pixel.

The software mask is a filter algorithm, which picks exclusively non-charge-sharing photon signals. In particular, the algorithm is applied to the data of the unmasked pixel. For each pixel, the algorithm compares the signal registered in the pixel itself *versus* the signal detected by its eight neighbor pixels. Only if at least 90% of the charge generated by a photon event is deposited in the center pixel (*i.e.* no charge sharing) is the signal included in the spectrum. This algorithm represents the software equivalent to the charge-sharing suppression mask which is introduced in this paper.

The cluster-finding algorithm sums the signal collected by a cluster of 2 × 2 pixels (alternatively, this could be set to 3 × 3 pixels) in order to collect the full charge deposited by a photon event and is described by Bergamaschi *et al.* (2015 ▸). Briefly, the algorithm adds up the charge within the 4 (or 9) pixel cluster whenever the signal of a single pixel or the full cluster signal are above a threshold of typically 5 × r.m.s. baseline. The algorithm can only be applied in the low-count-rate regime where on average less than one photon impinges per 2 × 2 (or 3 × 3) pixels cluster. The 2 × 2 clusters are chosen for this analysis since they remove charge sharing and the 3 × 3 pixel clusters degrade the energy resolution even more due to the summing of the electronic noise (Bergamaschi *et al.*, 2015 ▸).

Fig. 7(*b*) ▸ displays single-pixel Mo fluorescence spectra acquired with the same X-ray tube setup (60 kV, 25 mA) and the same detector settings (663 kframes). A spectrum with the mask (red), one without the mask (black), a spectrum obtained with a ‘software mask’ (blue) and a spectrum computed from a ‘cluster finding’ algorithm (gray) are compared. The effect of the different masks becomes evident in the Mo spectra, *i.e.* the presence of either mask clearly suppresses the charge-sharing contribution in the energy range from 750 eV to 17 keV [Fig. 7(*b*) ▸, green dashed box]. The effect of the charge-sharing suppression on the detection efficiency may be evaluated based on these spectra since the spectra are recorded under homogeneous illumination in the same measurement. The *K*_α_(Mo) peaks (Fig. 7*c* ▸) suggest a detection efficiency of about 14% for the masked pixel, of 23% for the software mask pixel and of 129% for the cluster-finding algorithm pixel as compared with a pixel situated outside the mask (integral of the peak from 17–18 keV). The increased efficiency of the cluster-finding algorithm as compared with the ‘no mask’ pixel is to be expected due to the summation of charge over a cluster of multiple pixels which recovers otherwise ‘lost’ counts. The detection efficiency for the total photon signal, determined by comparing the integral of the spectra in the energy range from 8.5 keV {= *E*[*K*_α_(Mo)]/2} to 21 keV, is about 9% for the masked, 16% for the software mask and 100% for the cluster finding pixel *versus* the ordinary pixel. The difference between the observed efficiency of 9% for the tungsten mask *versus* the expected efficiency of 11% (§2.3) is most likely due to geometrical effects, *i.e.* parallax in the measurement geometry and the conical rather than cylindrical shape of the laser-drilled mask holes. The efficiency of 16% for the software mask suggests that the algorithm corresponds to a mask with a hole diameter of about 34 µm.

Figs. 7(*b*) and 7(*c*) ▸ show the spectra obtained with the JUNGFRAU 0.4 detector without the mask, with the mask, with the software mask and using the cluster-finding algorithm. It should be noted that both the hardware and the software mask remove the charge-sharing contribution to the spectra at the cost of detection efficiency. In this measurement, the software mask shows a slightly higher detection efficiency as compared with the hardware mask. The cluster-finding algorithm removes the charge sharing well and is equally efficient as the unmasked geometry.

Generally, the choice between a hardware and a software mask/algorithm should be motivated by the experimental situation. Advantages for a hardware mask are the clean suppression of photons in the charge-sharing region of the pixels independent of the photon rate. The hardware mask requires the placement of the mask, introduces self-fluorescence of the mask material and causes a reduced detection efficiency. A software mask or cluster-finding algorithm are simple to implement, equally and more efficient than a hardware mask, respectively, and can be applied to existing ‘no mask’ data if the experiment is conducted in the single-photon regime, *i.e.* at sufficiently low photon rates.

### 5.2. Multi-color imaging at the synchrotron   

X-ray fluorescence (XRF) scanning imaging experiments are performed with JUNGFRAU 0.4 (with and without mask) on a temporary scanning microprobe station at the Nano­scopium beamline (Somogyi *et al.*, 2015 ▸) at the SOLEIL Synchrotron, Gif-sur-Yvette Cedex, France. The 13.3 keV X-ray beam is focused by a Fresnel zone plate (FZP) into a few µm (horizontal) × 0.5 µm (vertical) FWHM beam with an intensity of about 10^9^ photons s^−1^.

JUNGFRAU 0.4 is integrated in a Flyscan-like architecture (Medjoubi *et al.*, 2013 ▸) to perform fast XRF continuous-scanning imaging. In particular, a hardware trigger signal synchronizes the acquisition of the detector and the readout of the encoded motor positions. The registered signal on the detector and the motor encoder positions are read out simultaneously at each position of the continuous scan with an exposure/dwell time determined by the period of the trigger signal.

The XRF sample is a calibration chart, which features a SOLEIL logo (250 µm wide and 75 µm high) fabricated from Ni and Au. The sample is mounted at approximately 45° with respect to the beam direction, while the JUNGFRAU 0.4 detector is placed at a distance of about 5 cm from the sample, perpendicular to the incident beam in the horizontal plane to minimize the detection of elastic X-ray scattering from the sample. The SOLEIL logo is scanned continuously in the horizontal direction and in steps in the vertical direction. 1000 positions of 750 nm per line and 160 lines spaced by 300 nm are acquired. The detector exposure time and dwell time are 400 µs and 20 ms (50 Hz), respectively.

**Figure 8:**
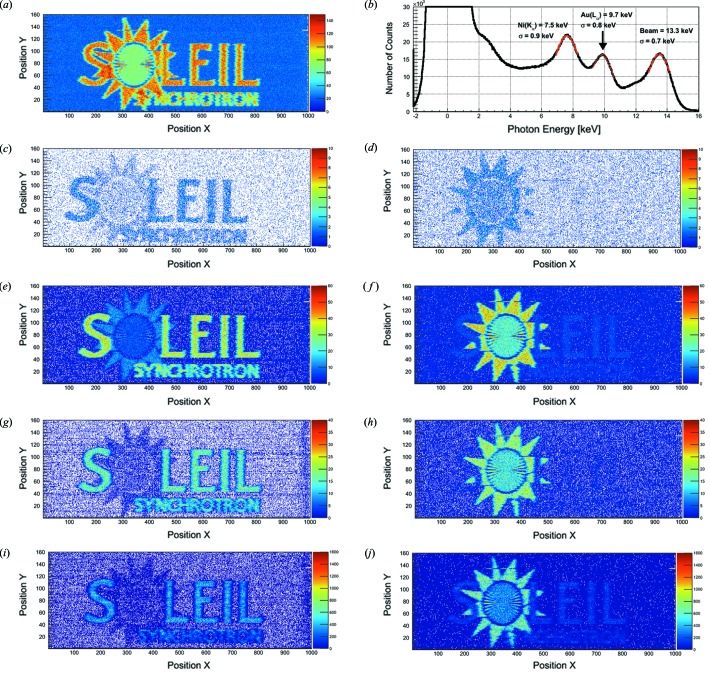
Flyscan images and spectrum of a fluorescence imaging target. The letters of ‘SOLEIL’ are made of Ni, while the material of the sun (with a Siemens resolution star at the center) is Au. (*a*) Full photon image of the Flyscan. (*b*) Gain-corrected integral photon spectrum of the entire Flyscan. (*c*)–(*j*) Two-color photon images where the Ni letters (left column) and the Au sun (right column) are selected by photon energy for JUNGFRAU 0.4 in combination with the mask (*c*, *d*), without the mask (*e*, *f*), the software mask (*g*, *h*) and the cluster-finding algorithm (*i*, *j*).

Fig. 8(*a*) ▸ displays a reconstructed full photon image where the photon signal recorded by JUNGFRAU 0.4 (without the mask) is integrated per Flyscan position and registered in the according position in the Flyscan image. This uncorrected image reveals a slightly higher spatial resolution in the *x*- than in the *y*-direction which reflects the oval distribution of the focused beam at the focal plane. The resolution of the scan is slightly degraded by the fact that no motor stage position correction is applied to the presented data. This will be remedied in future experiments.

Figs. 8(*c*)–8(*j*) ▸ show different two-color images (Ni and Au) of Flyscan measurements with JUNGFRAU 0.4 in combination with the mask, without the mask, with the software mask and the cluster-finding algorithm (each configuration plotted on the same scale). The two-color images are obtained by energy-selecting the Ni and Au photon signal, respectively, and plotting those in the Flyscan map.

An evaluation of the two-color images shows that JUNGFRAU 0.4 in all configurations (with the mask, without the mask, with the software mask, with the cluster finding algorithm) separates the different fluorescence components (Ni and Au) in the image. However, the mask compared with the software mask reduces the detection efficiency as expected and discussed in §5.1. In this experimental configuration, the software mask separates the Ni and the Au signal best. The cluster-finding algorithm, JUNGFRAU 0.4 without the mask and with the mask separate the Ni and Au signal less cleanly (see §S1 of the supporting information). Whether software rather than hardware charge-sharing suppression should be chosen for future Flyscan measurements might depend on the signal level and the resulting requirements for the detection efficiency.

The gain-corrected integral spectrum of the Flyscan (Fig. 8*b* ▸) as acquired with JUNGFRAU 0.4 demonstrates an energy resolution Δ*E*/*E* = 0.12 for the Ni *K*_α_ lines (FWHM = 2.1 keV), Δ*E*/*E* = 0.08 for the *L*_α_ lines of Au (FWHM = 1.8 keV) and Δ*E*/*E* = 0.05 for the photons from the synchrotron beam (FWHM = 1.7 keV).

### 5.3. Estimated rate capabilities   

The rate capability is an important characteristic of an energy-dispersive detection system. The rate capability of the JUNGFRAU 0.4 prototype is estimated and compared with the rates obtainable with SDDs, which are established energy-dispersive detection systems for photon detection, which typically possess an energy resolution of about <160 eV (FWHM). Present day state-of-the-art SDDs can handle up to about 3 × 10^6^ counts s^−1^ (Ketek GmbH, 2015 ▸; Amptek Inc., 2015 ▸). In the JUNGFRAU systems, the photon rate capability depends on the readout rate of the chip and on the number of pixels, which can be occupied per readout frame. The readout speed of the JUNGFRAU 0.4 prototype chip is 40 MHz with a single serial output. Hence, if JUNGFRAU 0.4 is combined with the charge-sharing suppression mask, every pixel can be used independently and about 4 × 10^7^ counts s^−1^ can be detected and their energy information can be recovered. For JUNGFRAU 0.4 without the mask and in combination with the software mask, at least nine pixels need to be allocated per photon event, *i.e.* the center pixel and its neighbors, hence the achievable photon count rate is reduced to 4 × 10^6^ counts s^−1^. The cluster-finding algorithm requires isolated single-photon events and hence at least 16 pixels, *i.e.* the cluster of 2 × 2 pixels and its neighbors, per photon event. Hence, the photon rate is reduced to about 2.5 × 10^6^ counts s^−1^. Future detector systems might enable higher readout rates, *e.g.* an increase by a factor of 10 to 40 is easily achievable by parallelizing the outputs. But, already at present, the photon rate capabilities of JUNGFRAU 0.4 are highly competitive with respect to SDDs.

## 6. Conclusions and outlook   

JUNGFRAU is a charge-integrating hybrid pixel detector which is designed for photon science at X-ray free-electron lasers, in particular SwissFEL, and synchrotrons. JUNGFRAU 0.4 is a very low noise variant of JUNGFRAU which is being tested for its potential as a low-energy or as an energy-dispersive detection system. The performance of JUNGFRAU 0.4 is evaluated also in combination with a charge-sharing suppression mask, a software mask and a cluster-finding algorithm.

Concerning the detection of X-rays at low energies, importantly JUNGFRAU 0.4 shows a r.m.s noise as low as 27 e^−^ (<100 eV). Photon detection down to 1.2 keV is shown and photon energies <1 keV should be accessible. Noise values of 170–180 e^−^ at an acquisition time of 2 ms suggest that the operation of such a detection system is feasible in the synchrotron environments. JUNGFRAU 0.4 has single-photon sensitivity and a good linearity. The system has sufficient dynamic range for many applications in the low-energy range, *e.g.* 49 × 1 keV photons, despite its fixed gain, where the introduction of automatic gain switching could allow the extension of the dynamic range in a future detector version. Points for further improvement towards low-energy detection include the separate optimization of the sensor and ASIC of this hybrid pixel detector for low-energy applications. In particular, the sensor entrance window is to be optimized for the energy range below 1.5 keV. The pixel circuit noise could be reduced even further by optimization of the gain, analog chain and preamplifier.

The capabilities of JUNGFRAU 0.4 as an energy-dispersive detection system are studied. JUNGFRAU 0.4 records multi-energy fluorescence spectra and images at an r.m.s. energy resolution of 20% (no mask) and 14% (with the mask) at 1.2 keV and of 5% at 13.3 keV. JUNGFRAU 0.4 spectra without the mask, with the mask, in combination with the software mask and the cluster-finding algorithm are compared and the detection efficiency is evaluated. Importantly, the photon rate capabilities of the JUNGFRAU 0.4 system are >10^6^ counts s^−1^ (up to 4 × 10^7^ counts s^−1^ for the masked assembly), which is highly competitive with respect to the photon rates achieved with present day SDDs. JUNGFRAU 0.4 needs to provide low noise levels as presented in this article and therefore requires high frame rates in excess of 100 kHz for future applications as an energy-dispersive detection system at synchrotron light sources. Such frame rates result in dead-time-free acquisition times of the order of 10 µs and can easily be achieved for small systems (as the presented prototype) by parallelization of the chip readout. Attention should be paid to the mask geometry and materials used for the charge-sharing suppression version of JUNGFRAU 0.4. An appropriate material choice and high-purity materials reduce the fluorescence background from the system itself. The size and shape of the laser-drilled hole mask should also be investigated and tuned to the JUNGFRAU 0.4 pixel.

In conclusion, JUNGFRAU 0.4 represents a possible starting point for a general purpose low-energy detector development or for a dedicated energy-dispersive system for spectroscopic measurements/imaging, which both could advance the experimental capabilities of photon science at synchrotron and free-electron laser research facilities.

## Supplementary Material

X-ray color separation with JUNGFRAU 0.4. DOI: 10.1107/S1600577515023541/pp5080sup1.pdf

## Figures and Tables

**Table 1:** Noise overview for the JUNGFRAU 0.4 chip bump-bonded to a 500 µm silicon sensor for no filtering and extra filtering. The total r.m.s. noise as well as the noise contributions from the preamplifier, the correlated double sampling (CDS) stage and the chip readout noise are displayed in units of electrons and (photon) energy in eV. The corresponding confidence level for detection above 5σ r.m.s. noise is given

R.M.S. noise	No filter capacitor	Extra filter capacitor
Total (e^−^)	35 ± 8	27 ± 4
Preamplifier (e^−^)	31	21
CDS (e^−^)	11	12
Readout (e^−^)	12	12
Total (eV)	126 ± 29	97 ± 14
5σ total (eV)	630 ± 145	349 ± 70
