# Supplementary material for: Towards hybrid pixel detectors for energy-dispersive or soft X-ray photon science
Source: J Synchrotron Radiat. 2016 Feb 10;23(Pt 2):385–94. doi: 10.1107/S1600577515023541 (PMC5297903; doi:10.1107/S1600577515023541)
Supplement: Supplementary file 1 [file s-23-00385-sup1.pdf]

JOURNAL OF SYNCHROTRON RADIATION

Volume 23 (2016)

Supporting information for article:

Towards hybrid pixel detectors for energy-dispersive or soft X-ray photon science

J. H. Jungmann-Smith, A. Bergamaschi, M. Brückner, S. Cartier, R. Dinapoli, D. Greiffenberg, T. Huthwelker, D. Maliakal, D. Mayilyan, K. Medjoubi, D. Mezza, A. Mozzanica, M. Ramilli, Ch. Ruder, L. Schädler, B. Schmitt, X. Shi and G. Tinti

S1. X-ray color separation with JUNGFRAU 0.4

In section 5.2, an energy-dispersive Flyscan experiment on a fluorescence imaging target is presented. In particular, a two-color target composed of Au and Ni is imaged. The efficiency of the X-ray color separation by JUNGFRAU 0.4 in combination with the charge sharing suppression mask, without the mask, with the software mask and the cluster finding algorithm is evaluated from the two-colour images of Figure 8. It is evaluated how efficiently the two X-ray energies are separated in the images. Therefore, it is determined how much Ni contamination is found in the image of an Au region of the target and how much Au contamination is registered in the image of a Ni region of the target. These ratios are corrected for the background Ni and Au signal in regions outside the Ni letters or Au sun of the target, respectively. Figure S1 shows the regions of the target/image employed for this test. Table S1 displays the degree of contamination in the two-color separation. Clearly, the software mask achieves the best degree of separation, i.e. the contamination from the other color is 5% or less for this method. It is also possible to achieve a good separation with the cluster finding algorithm and the data from JUNGFRAU 0.4 without the mask (degree of contamination from < 1% to 29 %). In this experiment, the hardware mask achieves a degree of contamination of 6 % for the Au found in the Ni region, while the degree of contamination is 84 % for the Ni in the Au region (possibly due to statistical fluctuations on this very low count rate experiment with the mask).

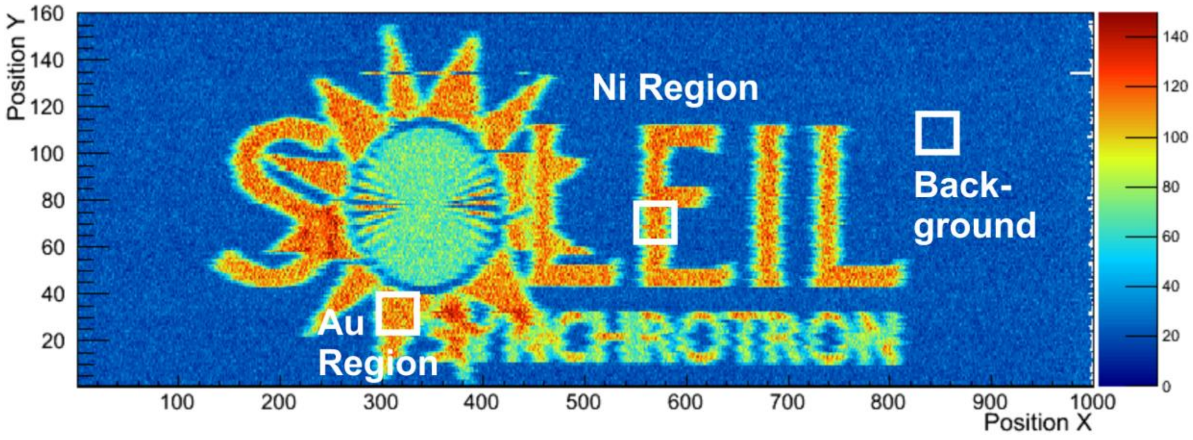

Figure S1 Full photon image of the Flyscan of the imaging target. The letters of the SOLEIL logo are composed of Ni, while the sun made of Au. The regions of the target/image used to evaluate quality of separation of the X-ray colors are indicated.

Table S1 Overview of the degree of contamination in the two-color imaging experiment for JUNGFRAU 0.4 in combination with the charge sharing suppression mask, without the mask, with the software mask and the cluster finding algorithm. The relative amount of the Ni contamination in an Au region, i.e. in the sun of the logo, is given by Ni(“Sun”)/Au(“Sun”), while the Au contamination in a Ni region, i. e. in the letter “E” is denoted by Au(“E”)/Ni(“E”).

	**Ni(“Sun”)/Au(“Sun”) [%]**	**Au(“E”)/Ni(“E”) [%]**
**Mask**	86	6
**No Mask**	27	8
**Software Mask**	5	3
**Cluster Finding Algorithm**	< 1	29
